# Pathologies digestives associées à la drépanocytose à Lubumbashi: aspects épidémiologiques et cliniques

**DOI:** 10.11604/pamj.2019.33.253.18017

**Published:** 2019-07-26

**Authors:** Manix Ilunga Banza, Jules Panda Mulefu, Lire Ipani Lire, Yannick Tietie Ben N'dwala, Israel Badypwyla Tshiamala, Vincent de Paul Kaoma Cabala

**Affiliations:** 1 Université de Lubumbashi, Faculté de Médecine, Département de Chirurgie, Cliniques Universitaires de Lubumbashi, Lubumbashi, République Démocratique du Congo

**Keywords:** Drépanocytose, pathologie digestive, fréquence, Sickle cell disease, digestive disease, frequency

## Abstract

**Introduction:**

la drépanocytose est une maladie génétique de transmission autosomique liée à une anomalie de structure de l'hémoglobine qui aboutit à la formation de l'hémoglobine S. Le but de notre étude est de colliger les cas de pathologies digestives rencontrées chez les drépanocytaires de Lubumbashi et d'en ressortir les caractéristiques épidémiologiques et cliniques.

**Méthodes:**

il s'agit d'une étude rétrospective, descriptive transversale réalisée au Centre de Recherche de la Drépanocytose de Lubumbashi. Elle a concerné les dossiers des patients suivi pour drépanocytose ayant présenté une pathologie digestive au cours de notre période de 3 ans (de janvier 2015 à décembre 2017). Le recueil des données s'est fait grâce à une fiche d'enquête comportant différents paramètres d'étude comprenant: l'âge, le sexe, le motif de consultation, le diagnostic, le type de crise vaso-occlusive, les examens paracliniques réalisés, le traitement à l'hydroxyurée.

**Résultats:**

nous avons colligé 206 dossiers (N=206) des patients drépanocytaires ayant fait une pathologie digestive sur un total de 403 dossiers examinés, ce qui représente une fréquence de 51,11% des pathologies digestives chez les drépanocytaires. Les 2 sexes sont représentés avec une légère prédominance féminine (51,94%) et un sexe ratio H/F: 0,92. La tranche d'âge la plus représentée est celle comprise entre 1 et 6 ans (32,52%), la moyenne d'âge: 11,8ans; écart-type: 21,9; âges extrêmes: 13 mois et 38 ans. Le motif de consultation est dominé par la fièvre (60,67%), la douleur abdominale (44,66%) et les troubles digestifs (30,09%). Les crises vaso-occlusives abdominales sont retrouvées chez 65 patients (31,55%) parmi lesquels 36 patients ont présenté 1 seule crise, 24 en ont présenté 2 et 5 patients en ont présenté 3. Les pathologies intestinales étaient présentes chez 121 patients (69,41%) dominées par la parasitose intestinale (retrouvée chez 58 patients dont l'examen des selles a mis en évidence 4 parasites: le Yersinia enterocolitis, l'entamoeba histolytica, le Giardia intestinalis et le clostridium difficile); les pathologies gastriques retrouvées chez 105 patients (50,97%) reparties en ulcère gastro-duodénal (45 patients) et gastrite (60 patients); la pathologie vésiculo-biliaire présente chez 40 patients (19,41%) comprenant la lithiase vésiculaire sans cholécystite 32 patients, la cholécystite lithiasique 5 patients et 3 cas de lithiase de la voie biliaire principale; 1 seul cas de pancréatite aigue diagnostiquée. Les pathologies associées les plus retrouvées dans notre étude sont respiratoires avec 169 cas (82,03%), oto-rhino-laryngologiques avec 157 cas (76,21%), les crises vaso-occlusives osseuses (146 cas soit 70,87%), pathologies uro-génitales avec 64 cas (31,06%) et le paludisme chez 51 patients (24,75%). Les atteintes spléniques et hépatiques ont constitué chacun 47 cas (22,81%) et 18 cas (8,73%). L'échographie a était demandé chez 79 patients mais seulement 31 d'entre-eux l'ont réalisé, faute de moyen financier car il coute sur place 20 dollars américains. En cas de splénomégalie cliniquement évidente, le corps de Jolly a été demandé chez 23 patients mais seulement 2 patients l'ont réalisé vu qu'il coute 10 dollars américains. L'hémogramme de routine fait de l'hémoglobine, hématocrite, bilan inflammatoire et la goutte épaisse a été réalisée chez tous nos patients mais le bilan hépatique, les examens des selles, des urines sont préconisés en fonction de la plainte. Sur nos 206 patients, 60 seulement d'entre eux étaient sous traitement à l'hydroxyurée (29,16%).

**Conclusion:**

les pathologies digestives sont fréquentes chez les drépanocytaires et représentent quasiment la moitié de l'effectif drépanocytaire. Malheureusement, la meilleure prise en charge reste butée à la pauvreté manifeste de la population limitants les examens paracliniques très utiles dans la pathologie digestive rencontrée chez le drépanocytaire.

## Abstract (en)

**Introduction:**

sickle cell disease is a genetic disease with autosomal inheritance associated with haemoglobin structure abnormality which causes the formation of hemoglobin S. The purpose of our study was to collect data on digestive diseases in patients with sickle cell disease in Lubumbashi and to highlight their epidemiological and clinical features.

**Methods:**

We conducted a retrospective, descriptive, cross-sectional study at the Research Center for Sickle Cell Disease in Lubumbashi. All the records of patients on follow-up for sickle cell disease with digestive disease during our 3-year period (January 2015 to December 2017) were analyzed. Data were collected using a survey taking into account different study parameters including: age, sex, the reason for consultation, diagnosis, the type of vaso-occlusive crisis, the paraclinical examinations made, hydroxyurea treatment.

**Results:**

out of a total of 403 medical records examined we found 206 cases (n=206) of sickle cell disease associated with digestive disease, accounting for a rate of 51,11% of patients with sickle cell disease who suffered from digestive diseases. Both sexes were represented with a slight female predominance (51.94%) and a sex ratio M/F of 0.92. The most represented age ranges 1-6 years (32.52%), the average age was 11.8 years; the standard deviation was 21.9; the extreme ages were 13 months and 38 years. The reason for consultation was dominated by fever (60,67%), abdominal pain (44.66%) and digestive disorders (30,09%). Vaso-occlusive abdominal crises were found in 65 patients (31.55%) among whom 36 had only 1 crisis, 24 had 2 crises and 5 had 3 crises. Intestinal diseases were found in 121 patients (69,41%) dominated by intestinal parasites (found in 58 patients whose collection of stool samples showed 4 parasites: Yersinia enterocolitis, Entamoeba histolytica, Giardia intestinalis and Clostridium difficile). Gastric diseases were found in 105 patients ( 50,97%) divided into peptic ulcer (45 patients) and gastritis (60 patients); biliary vesicular disease was found in 40 patients (19.41%) including vesicular lithiasis without cholecystitis (32 patients), lithiasic cholecystitis (5 patients) and lithiasis in the main biliary tract (3 cases); there was 1 single case diagnosed with acute pancreatitis. The most common associated diseases in our study were respiratory diseases (169 cases;82,03%), oto-rhino-laryngological diseases (157 cases;76.21%), bony, vaso-occlusive crises (146 cases; 70,87%), urogenital diseases (64 cases; 31.06%) and malaria (51 patients; 24.75%). Hepatic diseases and diseases of the spleen were found in 18 cases (8.73%) and 47 cases (22,81%) respectively. Ultrasound was requested in 79 patients but only 31 of them underwent it because of the lack of financial means (it costs 20 U.S. dollars). In the case of clinically obvious splenomegaly, the search for Howell-Jolly bodies was requested in 23 patients but it was only performed in 2 patients because it costs 10 U.S. dollars). Routine blood count, hemoglobin, hematocrit, inflammatory assessment and thick drop examination were performed in all our patients but liver assessment, tests done on stool samples, urine test were recommended based on patient's complaint. Out of 206 patients, only 60 were under hydroxyurea treatment (29,16%).

**Conclusion:**

digestive diseases are common in patients with sickle cell disease and account for almost half of patients with diagnosed sickle cell disease. Unfortunately, best management is limited by poverty leading to less very useful paraclinical examinations in patients with digestive diseases resulting from sickle cell disease.

## Introduction

La drépanocytose est une maladie génétique de transmission autosomique liée à une anomalie de structure de l'hémoglobine qui aboutit à la formation de l'hémoglobine S. C'est une des maladies héréditaires récessives les plus fréquentes à travers le monde et en Afrique subsaharienne [1, 2]. Selon l'Organisation Mondiale de la Santé, 7% de la population est porteur du gène anormal de globine et dans certaines régions du monde jusqu'à 1% des nouveau-nés sont atteints d'une pathologie de l'hémoglobine. Cette pathologie est très fréquente dans le monde mais surtout dans la population d'origine africaine subsaharienne, Amérique, les Antilles, d'inde et du moyen orient ainsi que le bassin méditerranéen [3]. L'incidence annuelle est estimée à 305 800 drépanocytaires naissant dans le monde entier dont 80% se trouve en Afrique [4]. Le dépistage des nouveau-nés, le suivi clinique systématique et la prévention du sepsis et des défaillances multi-viscérales a contribué à augmenter l'espérance de vie chez les drépanocytaires dans plusieurs pays; cependant en ressources limités ou la majorité d'enfants drépanocytaires naissent, nombreux continuent à mourir dans leur jeune enfance, souvent non diagnostiqué et ce, par manque de programme de détection précoce et de prise en charge [5]. Globalement, la drépanocytose affecte 300 000 enfants chaque année, la plus grande prévalence dans les contrées où sévit la malaria de façon endémique comme le moyen orient, l'Afrique et le sud de l'Asie. C'est aussi estimé que dans plusieurs pays africains, 10 à 40% de la population est porteur du trait drépanocytaire [6]. En 2010, le centre mondial de contrôle des maladies avec le programme en charge de la surveillance des nouveau-nés lequel est en charge de la drépanocytose a rapporté que l'incidence de la drépanocytose était de 73,1 cas pour 1000 enfants noirs, de 3,0 cas pour 1000 enfants blancs et de 2,7 pour 1000 enfants asiatiques, hawaïens, ou autres enfants des îles enregistrées [7].

Aux USA, elle est très prévalent, affectant environ 100 000 américains [8]; C'est la première maladie dépistée en néonatologie avec 1 nouveau-né atteint sur 1900 naissances, et il y a 90 000 à 100 000 patients atteints du syndrome drépanocytaire majeur aux Etats-Unis d'Amériques [9]. En République Démocratique du Congo, les données épidémiologiques récentes ont montré que 2% des nouveau-nés sont homozygotes pour l'hémoglobine S et environ 40 000 naissances d'enfants drépanocytaires sont estimés chaque année, tandis que dans la population adulte le portage du trait drépanocytaire s'élève à environ 25% [10, 11] mais la maladie reste peu connue avec pour conséquence une forte mortalité dans un pays à ressources limitées [12, 13]. Dans une publication parue en 2016 dans american journal of haematology, les auteurs célèbrent le 40^ème^ anniversaire du *sickle cell disease* program, la prise en charge qui, en 40 ans, a permis de transformer cette maladie pédiatrique, mortelle dans les années 1970, en une maladie chronique de l'adulte de nos jours [9].

Il s'agit d'une maladie dont l'évolution est émaillée de multiples complications qui en font toute la gravité [14]. On y note notamment des manifestations cliniques telles qu'une anémie hémolytique chronique, des phénomènes vaso-occlusifs, une susceptibilité extrêmes aux infections, la douleur, les manifestations pulmonaires, cardiovasculaires [15], des complications oto-rhino-laryngologiques [16]; rénales [17], les manifestations neurologiques [18, 19] et les manifestations digestives qui feront l'objet de ce présent travail. La drépanocytose peut toucher plusieurs systèmes dont le tractus gastro-intestinal et les manifestations sont habituellement secondaire à l'infarctus des petits vaisseaux et à l'occlusion de la microcirculation. L'ischémie se manifestant par une crise abdominale avec douleur sévère pouvant traduire une maladie ulcéreuse, une pancréatite et rarement une ischémie intestinale [20]. Les patients avec drépanocytose ont une variété des pathologies gastro-intestinales incluant les calculs biliaires, hépatite, la boue biliaire, hépatomégalie, crises douloureuses, cirrhose avec une variété d'étiologie. Ces entités et d'autres relatives à l'atteinte gastro-intestinale en cas de drépanocytose sont décrit avec insistance d'après leurs signes morphologiques et biochimiques [21]. Les calculs biliaires sont une complication chronique très fréquentes [22] et reconnus depuis longtemps comme complication de la drépanocytose. Une étude échographique menée au Royaume uni a montré que plus de la moitié des homozygotes SS âgé de 10 à 65 ans avait un calcul biliaire [23], au Jamaïque, 13% pour la cholélithiase [24]. La majorité des patients drépanocytaires vivent dans des pays sous-développés où les maladies parasitaires endémiques sont fréquentes et cela peut exacerber leur état d'anémie chronique [25], l'administration de l'hydroxyurée et la transfusion sont fortement recommandées chez plusieurs patients avec anémie falciforme [15]; la transfusion doit être donnée pour corriger l'anémie avec un taux d'hémoglobine inférieur à 5 g/dl à cause de l'hémoglobine S qui a une faible affinité à l'oxygène et assure une bonne oxygénation malgré l'anémie [26].

## Méthodes

Il s'est agit d'une étude descriptive et rétrospective étalée sur une période de 3 ans soit de janvier 2015 à décembre 2017. Elle a été réalisée au centre de recherche de la drépanocytose de Lubumbashi. Ce centre localisé à Lubumbashi est un établissement public à caractère scientifique et technique créé en novembre 1982 à Kinshasa; l'ouverture à Lubumbashi s'est faite en 2011 par l'arrêté ministériel. Il a une double mission de prendre en charge la drépanocytose et d'effectuer les recherches pour améliorer les traitements propres pour la drépanocytose. La récolte des données s'est faite grâce à une fiche d'enquête anonyme ayant concerné les dossiers des patients drépanocytaires de tout âge et de tout sexe ayant consulté le centre pour une pathologie digestive diagnostiquée durant notre période d'étude allant de janvier 2015 à décembre 2017 soit une période de 3 ans. Ainsi les paramètres d'étude ont été: l'âge; le sexe, le motif de consultation, les signes objectifs, le diagnostic clinique, les examens paracliniques réalisés, les pathologies associées, le traitement à l'hydroxyurée. Ainsi ont été exclu de notre étude tout patient n'ayant pas développé une pathologie digestive au cours de notre période d'étude et tout celui qui ne fait pas parti de notre période d'étude. Ainsi 403 dossiers des patients ont été examiné parmi lesquels 206 avaient connu une pathologie digestive et ont été retenu pour la présente étude; ceci a donc constitué la taille de notre échantillon. Les données colligés ont été saisies et analysées avec Word et Excel 2010; Epi-info 3.2.

## Résultats

Nous avons colligé 206 cas de pathologies digestives sur un total de 403 dossiers examinés durant les 3 années de notre période d'étude soit une fréquence de 51,77% des cas. La tranche d'âge la plus touchée est celle de 1 à 6 ans avec 32,5% des cas suivi de celle de 7-11 ans (21,4%) et 12-16 ans (20,9% des cas) (Tableau 1). L'âge moyen est de 11,8±21,9 ans. Le sexe féminin prédomine avec 51,95% des cas contre 48,05% pour les hommes (Tableau 1); sexe ratio Homme/femme: 0,92 Les motifs de consultation sont multiples (Tableau 2); on y retrouve la fièvre chez 125 patients soit 60,67%, la douleur abdominale retrouvée chez 92 patients (44,66%), les troubles digestifs retrouvés chez 62 patients ( 30,06%), le ballonnement abdominal chez 10 patients (4,85%). Seulement 116 patients sur les 206 (56,31%) avaient des signes digestifs alors que 90 patients (43,68%) n'avaient pas de plaintes digestives malgré la confirmation diagnostique de la pathologie digestive par les examens paracliniques de routine chez le drépanocytaire. En fonction des diagnostics, les crises vaso-occlusives abdominales ont représenté 65 cas (31,55%); la pathologie digestive intestinale a représenté 121 cas (58,73%) reparti en parasitose intestinale (58 cas soit 28,15%), troubles digestifs (55 cas soit 26,69%), colopathie fonctionnelle (5 cas soit 2,42%), hernie inguinale (22 cas soit 10,67%) et appendicite (1 cas soit 0,48%); la pathologie gastrique 105 cas (50,97%) reparti en gastrite (60 cas soit 29,12%) et ulcère gastroduodénal (45 cas soit 21,84%); la pathologie vésiculaire retrouvé chez 40 patients (19,41%) repartis en lithiase vésiculaire (32 cas soit 15,53%), cholécystite (5 cas soit 2,42%) et cholécystolithiase ( 3 cas soit 1,45%); les atteintes spléniques retrouvé chez 47 patients (22,81%) reparties en 27 cas de splénomégalie (13,10%), 14 cas d'asplénie fonctionnelle (6,79%), 4 cas de séquestration splénique (1,94%) et 2 cas d'hypersplénisme); et les atteintes hépatiques 18 cas (8,73%) répartis en 16 cas d'hépatomégalie (7,76%), la séquestration hépatique et l'hépatite virale B chacun 1 cas (0,48%) chacune.

**Tableau 1:** répartition des cas en fonction de l’âge et du sexe

Variables	Effectif	Pourcentage (%)
**Tranche d’âge (ans)**		
1-6	67	32,5
7-11	44	21,4
12-16	43	20,9
17-21	21	10,2
22-26	20	9,7
27-31	7	3,4
32-36	3	1,5
37-41	1	0,5
**Sexe**		
Masculin	99	48,1
Féminin	107	51,9

La tranche d’âge la plus touchée est celle de 1-6 ans avec 32,5% des cas; âge moyen: 11,8 ± 21,9 ans; âges extrêmes: 13 mois et 40 ans Le sexe féminin prédomine légèrement par rapport au masculin avec 51,9% des cas; le sexe ratio homme/femme: 0,92

**Tableau 2:** répartition des cas selon les motifs de consultation

Motif de consultation	Effectif	Pourcentage (%)
Aucune plainte	90	43,68
Douleur abdominale	92	44,66
Fièvre	125	60,67
Troubles digestifs	62	30,09
Ballonnement abdominal	10	4,85
Hématémèse	**9**	**4,36**

Chez 90 patients, aucune plainte n’était évoquée à la consultation tandis que la fièvre a été le motif de consultation le plus présent avec 60,67% et la douleur abdominale présente chez 44,67% des patients

La pancréatite aigue découverte chez 1 seul patient (0,48%) (Tableau 3). Les pathologies associées retrouvées sont les pathologies respiratoires (82,03%), les crises vaso occlusives osseuses (146 cas soit 70,87%), oto-rhino-laryngologiques (76,21%), le paludisme (51 cas soit 24,75%), les pathologies urogénitales (64 cas soit 31,06%), la nécrose aseptique (21 cas soit 10,19%), les crises hémolytiques aigues (13 cas soit 6,31%), les pathologies rénales (6 cas soit2,91%) et les cardiopathies (3 cas soit 1,45%) (Tableau 4). Les examens biologiques systématiquement demandés sont faits d'un bilan inflammatoire (globules blancs, vitesse de sédimentation, protéine C réactive, formule leucocytaire), du taux d'hémoglobine, d'une goutte épaisse. Ceux-ci ont été demandé chez tous nos 206 patients. D'autres examens biologiques sont demandés en fonction de la clinique, il s'agit des examens des selles à frais réalisés chez 79 patients (38,34%), la coproculture 15 cas (7,28%), le bilan infectieux urinaire fait d’examen cytobactériologique chez 6 patients (2,91%), des transaminases demandés chez 43 patients (20,87%), le corps de joly demandé chez 23 patients (11,16%), la sérologie à l'hépatite A chez 3 patients (1,45%), l'hépatite B chez 2 patients (2,90%), la ponction lombaire chez 80 patients (38,83%). L'échographie a été demandé chez 79 patients (38,34%) pour le diagnostic des pathologies telles que la lithiase vésiculaire, la cholécystite, la splénomégalie, l'hépatomégalie mais seulement 31 patients l'ont réalisé. De nos 206 patients, seulement 60 d'entre-eux étaient sous traitement à l'hydroxyurée soit 29,12% des cas.

**Tableau 3:** répartition des cas en fonction des diagnostics

Etiologies	Effectif	Pourcentage (%)
Crise Vaso-occlusive abdominale	65	31,55
Crise vaso-occlusive légère	52	25,24
Crise vaso-occlusive modérée	11	5,33
Crise vaso-occlusive sévère	2	0,97
Parasitose intestinale	58	28,15
Colopathie fonctionnelle	10	4,85
Appendicite	1	0,48
Gastrite	60	29,12
Ulcère gastroduodénal	45	21,84
Lithiase vésiculaire	32	15,53
Cholécystite	5	2,42
Cholécystolithiase	3	1,45
Splénomégalie	27	13,10
Asplénie fonctionnelle	14	6,79
Séquestration splénique	4	1,94
Hypersplénisme	2	0,97
Hépatomégalie	16	7,76
Hépatite	1	0,48
Séquestration hépatique	1	0,48
Hernie inguinale	22	10,67

Les crises vaso-occlusives abdominales ont représenté 31,55% (25,24% des crises légères, 5,33% des crises modérées et 0,97% des crises sévères), parasitoses intestinales (28,15%)

**Tableau 4:** répartition des cas en fonction des pathologies associées

Pathologies (Systèmes)	Effectif	Pourcentage %
Respiratoire	169	82,03
Oto-rhino-laryngologiques	157	76,21
Urogénitale	64	31,06
Système locomoteur	146	70,87
Cardiovasculaire	3	1,45
Paludisme	51	24,75

## Discussion

Notre étude portant sur les aspects épidémiologiques et cliniques des pathologies digestives chez les drépanocytaires a colligé 206 cas sur 403 dossiers des patients examinés. La fréquence de la pathologie digestive chez les drépanocytaires Lushois est de 51,11%. Sans donner des valeurs chiffrées de l'ensemble des pathologies digestives, les auteurs sont unanimes sur la fréquence élevée de celles-ci chez le drépanocytaire.

**Par rapport à la tranche d'âge:** la tranche d'âge la plus touchée dans notre série est celle comprise entre 1-6 ans avec 32,5% des cas avec une moyenne d'âge de 11,8 ans pour des âges extrêmes de 13 mois et 38 ans. A Ouagadougou, Solange Odilet Diarra Ye trouvent un âge moyen inférieur au notre respectivement de 7 ans et 7,9±3,87 ans, ceci s'expliquant par le fait que leur étude ne concernait que les enfants d'au plus 15 ans [27, 28] alors que la notre reprenait les patients de tout âge; La tranche d'âge la plus touchée est celle de 6-10 ans avec 41% [28].

**En fonction du sexe:** notre étude révèle une légère prédominance féminine avec un sexe ratio de 1,08 en faveur des femmes. Mais en rapport avec le sexe, les études sont contradictoires. Certaines montrent une prédominance masculine [28, 29]; d'autres une prédominance féminine [29-32]; d'autres encore une répartition égale entre les 2 sexes [33, 34]. Nous pensons que la prédominance de l'un ou l'autre genre est le fait du hasard.

**En rapport avec le motif de consultation:** les motifs de consultation chez les patients drépanocytaires avec pathologie digestive sont multiples. Les signes généraux sont au premier plan dominé par la fièvre dans notre série avec 60,67% des cas alors que Sonia [32] rapporte une fièvre dans 95,5% des cas, fréquence très supérieure à la notre; ceci est due au fait que son étude était centrée sur les infections chez les drépanocytaires. Seulement 116 patients sur 206 (56,31%) avaient des signes cliniques digestifs dominés par la douleur abdominale (44,66%) puis le ballonnement abdominal (4,85%) alors que 90 patients (43,69%) n'avaient pas de plaintes lors de la consultation. Sonia [32] rapporte 23,3% des douleurs abdominales, faible par rapport à la notre probablement pour la raison que son étude n'était pas centrée sur le tube digestif comme la notre.

**Par rapport au diagnostic étiologique:** en fonction des diagnostics, les crises vaso-occlusives abdominales ont représenté 65 cas (31,55%) dans notre série par contre Roger Dodo au bénin rapporte 12,7% pour les vaso-occlusives abdominales [35]; cette différence pourrait s'expliquer par le fait que son étude était uniquement centrée sur les urgences drépanocytaires alors que la notre était centrée sur les pathologies digestives urgentes ou non. Chez les patients avec crise vaso-occlusive, la crise hépatique aigue drépanocytaire est rapporté chez 10% des patients [36]; cliniquement il peut se présenter comme une crise de cholécystite avec fièvre, hypochondralgie, ictère [37].

La pathologie digestive intestinale a représenté 121 cas (58,73%) dans notre étude. Nous y avons trouvé la parasitose intestinale (58 cas soit 28,15%) dominé par le Yersinia enterocolitis (28 cas) et l'Entamoeba hystolitica (19 cas) et Giardia intestinalis (11cas), la colopathie fonctionnelle (60 cas soit 29,12%), entérite fébrile (2 cas soit 0,97%), la hernie inguinale (22 cas soit 10,67%) et l'appendicite (1 cas soit 0,48%); en Afrique subsaharienne, les auteurs rapportent des fréquences de 38,8% pour la Giardiase ainsi que 27,8% et 25,9% pour l'amibiase [25-33]. les parasites du tube digestif sont retrouvés dans toutes les tranche d'âge chez Sonia [32] et sont dominés par Gardia intestinalis (8 cas sur 17 ) et Entamoeba histolitica (4 cas sur 17) également des protozoaires comme dans notre série; ceci confirme bien les données rapportés par des auteurs que la majorité des patients drépanocytaires vivent dans les pays sous développés ou les maladies parasitaires endémiques sont fréquentes ce qui peut exacerber leur état d'anémie chronique [25]. Ceci pourrait s'avérer réel puis que nous avons trouvé le paludisme comme l'une des pathologies associées les plus fréquentes avec 24,75% des patients. Ce taux est légèrement faible par rapport à celui rapporté par Roger Dodo [35] de 27,5% des cas mais supérieur à celui trouvé par Sonia [32] de 16,5% et Mbika [38] de 18%. Les interactions entre drépanocytose et paludisme restent observées et la croyance selon laquelle les drépanocytaires sont protégés reste répandue [39]; ce qui prouve la controverse de cette croyance. Les patients hétérozygotes dont leur globules rouges contiennent l'hémoglobine A et S seraient fortement protégés de la malaria [40-41] mais la distribution globale et la fréquence de la beta mutation S reflète actuellement l'incidence historique de décès dus à la malaria [6]. En rapport avec les examens biologiques, ils sont demandés en fonction de la clinique, il s'agit des examens des selles à frais réalisés chez 79 patients (38,34%) alors que dans l'étude de Sonia [32] examen parasitologique des selles était réalisé chez 16,5% des patients, la coproculture 15 cas (7,28%) contre 13,5% chez sonia [32], le bilan infectieux urinaire fait d’examen cytobactériologique chez 6 patients (2,91%) contre 19,3% chez Sonia [32], des transaminases demandés chez 43 patients (20,87%), le corps de joly demandé chez 23 patients (11,16%), la sérologie à l'hépatite A chez 3 patients (1,45%), l'hépatite B chez 2 patients (2,90%), la ponction lombaire chez 80 patients (38,83%). La pathologie gastrique 105 cas (50,97%) reparti en gastrite (60 cas soit 29,12%) et ulcère gastroduodénal (45 cas soit 21,84%); ce taux semble fréquent par rapport à la population non drépanocytaire. Il n'ya pas de conclusion évidente suggérant que l'incidence d'ulcère peptique chez les drépanocytaires est élevée que dans la population générale malgré la nature dépressive de cette maladie [42], cependant les symptômes dyspeptiques sont fréquent chez les drépanocytaires [42]. Lee MG trouve à l'examen endoscopique digestive une anomalie chez 20 patients sur 51 qui présentaient une douleur abdominale reparties en 14 cas d'ulcère duodénal, 4 cas d'ulcère gastrique et 2 cas de gastrique [43]. L'étiologie de ces ulcères est associée à la réduction de la résistance de la muqueuse secondaire à une ischémie répétée au cours de la drépanocytose [44]. La pathologie vésiculaire retrouvée chez 40 patients (19,41%) repartis en lithiase vésiculaire (32 cas soit 15,53%), cholécystite (5 cas soit 2,42%) et cholécystolithiase (3 cas soit 1,45%); le système hépatobiliaire est l'un des organes abdominaux les plus touchés par la drépanocytose et les atteintes hépatiques sont observées dans 10 à 40% des cas de séries drépanocytaires [4]. La fréquence de la lithiase biliaire chez le drépanocytaire augmente avec l'âge et la sévérité de la maladie.

En Jamaïque, la prévalence est estimée à 12% dans le groupe d'enfants de 5-7 ans et de 23% dans le groupe d'enfants âgés de 11 à 13 ans [24]. Ce qui corrobore avec nos observations et ceci à cause de l'hémolyse chronique libérant les pigments biliaires ce qui modifie l'équilibre du triangle d'Admirant et Small causant ainsi des calculs biliaires. Les atteintes hépatiques 18 cas (8,73%) répartis en hépatomégalie (16 cas soit 7,76%), séquestration hépatique (1 cas soit 0,48%) et hépatite virale B (1 cas soit 0,48%) alors que Johnson [36] trouve 32% d'anomalies chroniques du foie à la biologie comprenant l'hépatite, congestion chronique passive du foie, maladie alcoolique du foie, maladie vasculaire du collagène, et la sarcoidose; il trouve 19% d'hépatite alors que Koskina [45] trouve 39% d'atteinte hépatique, 9 cas d'hépatomégalie douloureuse(22%), 8 cas de cholestase. La crise hépatique aigue du drépanocytaire est rapportée chez 10% des patients présentant une crise vaso-occlusive [46]; cliniquement il peut se présenter comme une crise de cholécystite avec fièvre, hypochondralgie, ictère [37]. Nos résultats faibles sur les atteintes hépatiques sont probablement sous-estimés à cause de limites financières de la paraclinique aussi bien échographique que biologique. Les atteintes spléniques retrouvé chez 47 patients (22,81%) reparties en splénomégalie (27 cas soit 13,10%), asplénie fonctionnelle (14 cas soit 6,79%), séquestration splénique(4 cas soit 1,94%) et hypersplénisme(2 cas soit 0,97%). Sonia [32] rapporte une fréquence de splénomégalie et d'hépatomégalie supérieure à la notre soit respectivement de 42,1% et 30,1%. Notre taux bas pourrait s'expliquer par le fait que sur 79 patients seulement 31 patients ont fait l'échographie qui n'était donc pas systématique, certains patients asymptomatiques n'ayant pas fait l'échographie pourrait avoir une splénomégalie infra-clinique. La rate est presque toujours affectée dans la drépanocytose selon Ellen C. avec des micro-infarctus dans les 36 premiers mois de vie entrainant une atrophie splénique, les maladies chroniques du foie peuvent être dues à l'hémosidérose et l'hépatite [46]. Nous pensons que les hépatomégalies trouvées chez nos patients étaient causées par de l'hémosidérose malheureusement la non subvention du centre de recherche limite les examens complémentaires à la charge de la famille elle-même. Nous n'avons trouvé que 1 seul cas sur 206 cas alors que Johnson trouve que l'hépatite aigue affecte 10% des patients admis pour crise douloureuse abdominale [36]. Seulement 60 patients sur 206 étaient sous traitement à l'hydroxyurée soit 29,12%. L'hydroxyurée réduirait les complications de la drépanocytose, telles que les crises douloureuses [47]. Le traitement par l'hydroxyurée a été observé avec corrélation statistiquement significative à la réduction du taux d'hémoglobine S, avec une élévation de l'hémoglobine fœtale [48].

## Conclusion

Les drépanocytaires sont en proie à des pathologies digestives; notre étude a révélé la fréquence élevée des pathologies digestives chez le drépanocytaire de 51,1%. La clinique est dominée par la fièvre, la douleur abdominale et les troubles digestifs mais parfois le patient est asymptomatique, la découverte de la pathologie digestive se faisant par les examens de routine, ou de surveillance. Malheureusement le coût élevé de certains examens paracliniques limite la meilleure prise en charge dans une population avec une pauvreté manifeste.

### Etat des connaissances actuelles sur le sujet

- La drépanocytose est l'affection génétique la plus répandue dans le monde;
- Elle est surtout localisée en Afrique subsaharienne;
- Les pathologies digestives sont fréquentes chez le drépanocytaire.

### Contribution de notre étude à la connaissance

- La fréquence objective des pathologies digestives chez le drépanocytaires de Lubumbashi;
- Les caractéristiques épidémiologiques et cliniques des pathologies digestives des drépanocytaires;
- Les limites de la meilleure prise en charge de ces pathologies digestives.

## Conflits d’intérêts

Les auteurs ne déclarent aucun conflit d'intérêts.
